# Pharmacokinetics of repeated, high-dose fentanyl administration in rats

**DOI:** 10.1016/j.dmd.2026.100390

**Published:** 2026-08-17

**Authors:** David P. Obert, Autumn McKnite, Gwi H. Park, Kevin M. Watt, Christopher A. Reilly, Kathleen F. Vincent, Joseph E. Rower, Ken Solt

**Affiliations:** 1 Department of Anesthesiology and Intensive Care Medicine, School of Medicine and Health, Technical University of Munich, Munich, Germany; 2 Department of Anesthesiology, Mass General Brigham, Boston, Massachusetts; 3 Department of Anaesthesia, Harvard Medical School, Boston, Massachusetts; 4 Division of Clinical Pharmacology, Department of Pediatrics, University of Utah, Salt Lake City, Utah; 5 Department of Pharmacology and Toxicology, Center for Human Toxicology, University of Utah, Salt Lake City, Utah

**Keywords:** Fentanyl, Overdose, Opioid use disorder, Sex differences

## Abstract

Fentanyl is a potent synthetic opioid widely used as a recreational drug. It is primarily metabolized in the liver to the inactive compound norfentanyl, with only a small fraction excreted unchanged via the kidneys. However, it remains unclear whether repeated high-dose exposure alters its pharmacokinetics (PK) over time. This study evaluated the effects of repeated high-dose fentanyl administration on PK and assessed sex-related differences in metabolism. Male and female Sprague-Dawley rats (*n* = 19) received intravenous boluses of 100 *μ*g/kg fentanyl twice weekly for 5 weeks. After the 1st, 5th, and 10th doses, plasma concentrations of fentanyl and norfentanyl were measured using ultra-high-performance liquid chromatography–electrospray ionization–tandem mass spectrometry. PK data were analyzed using noncompartmental methods, followed by covariate analysis within a population PK model and a physiologically based PK model to identify underlying physiological factors. Noncompartmental analysis showed significantly lower C_max_ and longer t_1/2_ of norfentanyl in females than males. Population PK analysis using a two-compartment model confirmed slower fentanyl clearance and reduced norfentanyl formation in females. Repeated dosing led to decreased fentanyl clearance after the 10th exposure. In males, higher muscle volume was inversely correlated with total body clearance, whereas in females, fat volume correlated positively with total fentanyl exposure. Repeated high-dose fentanyl administration had only modest effects on PK, limited to reduced fentanyl clearance after the final exposure. Pronounced sex differences in fentanyl metabolism were observed with female animals clearing both fentanyl and norfentanyl more slowly and producing less norfentanyl than males.

**Significant Statement:**

In a rat model of repeated fentanyl exposure mimicking illicit use, PK changes over time were minimal, suggesting limited accumulation with intermittent dosing. In contrast, marked sex differences were observed, with female rats showing slower fentanyl clearance and reduced metabolite formation, highlighting sex-specific determinants of opioid metabolism with potential implications for interpreting toxicology findings and overdose risk.

## 1 Introduction

Fentanyl is a potent synthetic opioid that was first approved by the US Food and Drug Administration in 1968 for the induction of general anesthesia and the treatment of perioperative pain.^1^ Since then, its approval has been extended to treat complex pain conditions, as its analgesic potency is about 50–75 times higher than morphine depending on the context and route of administration.^2^, ^3^, ^4^, ^5^ Unfortunately, fentanyl has now become a commonly used recreational drug.^6^ This increase in illicit use coincides with a rising number of overdose deaths involving fentanyl.^7^^,^^8^ After intravenous fentanyl injection, the pharmacokinetic (PK) profile is described by rapid redistribution followed by slow terminal elimination.^9^ In humans, the terminal half-life (t_1/2_) ranges between 4 and 15 hours depending on age, whereas in rats, the t_1/2_ is significantly shorter (44–75 minutes) depending on the PK model.^10^, ^11^, ^12^ Due to its lipophilicity and large volume of distribution, fentanyl has a long context-sensitive half-life, increasing the risk of accumulation and overdose if injected repeatedly within a short period of time.^13^

Fentanyl is primarily metabolized in the liver via CYP3A4 to the inactive compound norfentanyl.^14^^,^^15^ Less than 1% is metabolized to hydroxyfentanyl, hydroxynorfentanyl, and despropionylfentanyl, and about 10% of the intact molecule is excreted renally and via feces.^16^ Fentanyl itself does not seem to alter CYP3A4 activity, but CYP3A4 inducers or inhibitors (eg, ketoconazole) influence fentanyl plasma concentrations.^17^^,^^18^ A recent study using a physiologically based PK (PBPK) model concluded that only 33% is metabolized to norfentanyl, whereas previous work suggested that it was greater than 80%.^15^^,^^16^^,^^19^^,^^20^ There is limited knowledge regarding the influence of repeated fentanyl exposures on its metabolism, and the role of PK in the development of tolerance. In chronic opioid users, the renal clearance is significantly protracted.^21^^,^^22^ Since there are sex-specific differences in cytochrome P450 expression, fentanyl metabolism may differ between males and females.^23^ However, most studies evaluating sex differences in opioid pharmacology have focused on pharmacodynamic (PD) and not PK parameters.^24^^,^^25^ Even though there are previous studies investigating sex differences in morphine metabolism and antinociception in rats, these findings cannot be extrapolated to fentanyl because morphine only undergoes phase II metabolism.^26^^,^^27^

Therefore, in this study, we evaluated sex differences in fentanyl metabolism and the effect of repeated high-dose fentanyl exposures on PK. We measured both fentanyl and norfentanyl plasma concentrations in an established rat model of repeated overdose to mimic illicit fentanyl use.^28^ Collected concentration data were then analyzed using multiple PK approaches to assess the influence of sex and repeated exposures on fentanyl PK.

## 2 Materials and methods

The Massachusetts General Hospital Institutional Animal Care and Use Committee approved all experimental procedures described in this study. Animals were treated in accordance with the Guide for the Care and Use of Laboratory Animals, and all efforts were made to reduce the number of animals and to minimize suffering.^29^ The report of the study follows the ARRIVE guidelines.^30^

### 2.1 Animals

Animals were purchased from Charles River Laboratories with an indwelling arterial catheter in the femoral artery connected to a vascular access button. Catheters were maintained by flushing weekly with heparinized glycerol (#HGS-10, SAI Infusion Technologies). Adult male and female Sprague-Dawley rats (280–380 g) were single housed to avoid damage to the vascular access buttons by cage mates and had access to food and water ad libitum. They were kept on a 12-hour light on/light off cycle (7am/7 pm) and a temperature of 22 °C. After arriving at the animal facility, the animals had at least 1 week to acclimatize before the start of the experiment.

### 2.2 Experimental design

The main purpose of this study was to investigate the influence of repeated high doses of fentanyl on PK. Additionally, we aimed to evaluate the influence of sex on these parameters. Because the literature on these topics is limited, we could not perform a proper sample size calculation and therefore relied on group sizes previously used to assess sex differences in opioid tolerance and PK in rats.^26^^,^^31^ The group sizes ranged from 3 to 8 animals, so we included 6–7 animals per sex in our study.

We identified 100 *μ*g/kg body weight as an intravenous dose in rats that reliably induced prolonged unconsciousness (assessed by the loss of righting reflex) mimicking human overdose. This dose was previously used in nonventilated rats to evaluate the effect of fentanyl on respiratory drive, and no fatalities were reported.^32^ Since fentanyl is commonly administered intravenously, we established a tail vein catheter before each fentanyl injection.^33^ To place the catheter, we put the animals in an acrylic chamber and induced general anesthesia with isoflurane (5 Vol.-%). After loss of righting reflex, animals were transferred to a heating pad and anesthesia was maintained through continuous isoflurane inhalation via face mask (2–2.5 Vol.-%). A 24-gauge intravenous catheter was inserted in the tail vein, flushed with saline, and secured with tape. Animals were transferred to the home cage and had at least 45 minutes to recover from the procedure before fentanyl was administered.

Based on the results of a previous study investigating the effect of fentanyl tolerance on respiratory depression, we injected the animals twice a week with at least 72 hours between exposures to ensure full washout of fentanyl and to provide sufficient time to recover.^34^ All fentanyl injections were administered between 10 am and 12 pm.

### 2.3 Sample collection

Samples were collected from the arterial catheter after the 1st, 5th, and 10th injections, as we observed that the time to return of righting reflex was longest after the first exposure, shortest after the 5th exposure, and then gradually increased again.^28^ For 4 animals, samples were collected after the 11th injection, as blood had already been drawn from these animals at the 10th injection for blood gas analysis, in line with the 3Rs principles to minimize animal use. Blood was drawn 3, 15, 30, 60, 180, and 360 minutes after the injection based on previous studies investigating the PK of fentanyl.^11^ Animals that died before the collection of the 360-minute sample were excluded from analysis. Accounting for the arterial catheter’s dead volume of approximately 0.09 mL, 0.15 mL of blood was first drawn and discarded. For analysis, a total of 0.30 mL of blood was drawn using a 1-mL syringe prerinsed with ethylenediaminetetraacetic acid (EDTA). Afterwards, the arterial catheter was flushed with 0.25 mL of 5 IU/mL heparin/saline solution (Shenzhen Techdow Pharmaceutical Co). To prevent clotting and to maintain patency of the arterial catheter, it was locked with 0.1 mL of heparin/glycerol solution (500 IU/mL) after the 60, 180, and 360-minute blood draws. The blood was transferred into an EDTA tube (#B-D365974Z, Medline Industries Inc), which was immediately placed on ice. The sample was centrifuged for 15 minutes at 1200*g* at 4 °C. After centrifugation, plasma was isolated and stored at −80 °C.

### 2.4 Sample analysis

Quantifying fentanyl and norfentanyl concentrations in rat plasma (EDTA as anticoagulant) used protein precipitation followed by ultra-high-performance liquid chromatography (UHPLC)- electrospray ionization (ESI)-tandem mass spectrometry (UHPLC-ESI-MS/MS). Fentanyl-d5 and norfentanyl-d5 were used as the internal standards. All reference materials were obtained from Cerilliant. A 50 *μ*L volume of the calibration standards, quality controls (QCs), blanks, and samples was aliquoted, followed by the addition of 50 *μ*L of internal standard solution (1 ng/mL). Cold acetonitrile (500 *μ*L) was added to precipitate plasma proteins, followed by centrifugation to separate the proteins, and then 500 *μ*L of the supernatant was transferred to a fresh sample tube. The supernatant was evaporated to dryness, and the extract reconstituted in 100 *μ*L of 10:90 methanol:0.1% aqueous formic acid. After mixing, the reconstituted extracts were transferred to autosampler vials for analysis by UHPLC-ESI-MS/MS. The UHPLC system was a ThermoScientific Vanquish UHPLC autosampler and pumping system interfaced with a ThermoScientific TSQ Quantis Plus triple quadrupole mass spectrometer. Chromatographic separation was performed on a Phenomenex Kinetex Biphenyl column (2.1 × 50 mm, 1.7-*μ*m particle size) using gradient elution flowing at 0.3 mL/min. The mobile phases were (A) 0.1% aqueous formic acid and (B) methanol, initially equilibrated at 10% B. The mobile phase composition was linearly increased to 100% B at 2.5 minutes, where it was held for 1 minute before being returned to and equilibrated at initial conditions. The MS/MS was operated in the selected reaction monitoring (SRM) mode under positive ESI conditions. For fentanyl, fentanyl-d5, norfentanyl, and norfentanyl-d5, the SRM transitions were mass/charge (collision energy) 337.2–188.2 (23 eV), 342.2–188.2 (23 eV), 233.2–84.2 (19 eV), and m/z 238.2–84.2 (19 eV), respectively. Calibrators were prepared for each analytical batch in rat plasma at nominal fentanyl and norfentanyl concentrations between 0.01 and 10 ng/mL. Samples determined to have concentrations >10 ng/mL were reassayed with either a 10- or 20-fold dilution into the range of the assay. A double blank (matrix without internal standard) and a zero sample (matrix with internal standard) were prepared for each run in the same lot of rat plasma used for the calibrators. QC samples were also prepared in rat plasma at nominal fentanyl/norfentanyl concentrations of 0.03, 0.2, 2, and 8 ng/mL. Interday accuracy (as % difference from nominal concentration) across all QC concentrations was within ±10.3% for fentanyl and ±4.6% for norfentanyl, whereas interday precision (as %CV) was within 4.9% for fentanyl and 5.2% for norfentanyl.

### 2.5 PK analysis

Multiple approaches for PK analysis were used to assess the role of sex and repeated exposures on the PK of fentanyl. First, fentanyl and norfentanyl concentration data were analyzed using a noncompartmental approach using Phoenix WinNonlin v8.3.2 (Certara Inc). Elimination slopes were best fit by the software, and the linear trapezoidal-linear interpolation method was used for calculations. Statistical differences between the calculated PK parameters (area under the concentration-time curve to infinity [AUC_∞_]), maximum concentration (C_max_), and half-life (t_1/2_)) were assessed using two-way ANOVA with a Tukey multiple comparison test in GraphPad Prism v11.0.1 (GraphPad Software). Next, we constructed a population PK model using NONMEM v7.5.1 (ICON plc) interfaced with Pirana v2.9.9 (Certara Inc) to determine covariates significantly associated with the PK of fentanyl. The first-order conditional estimation with interaction was used throughout model construction. Model selection was based on parsimony, objective function value (OFV, representing the −2 log likelihood), and diagnostic plots. The ADVAN13/TRANS-1 built-in model/parameterization subroutines were used to enable the construction of a parent-metabolite model using user-written differential equations. After the base model was established, covariates (including age (weeks), weight (grams), sex (male vs female), and injection number (1, 5, or 10) were tested on the model using the stepwise-covariate modeling (SCM) script available in Pirana. The SCM script tests covariate significance using forward inclusion (*P* < .05), followed by backward exclusion (*P* < .01), using a χ^2^ test with 1 degree of freedom. The final full model was then evaluated using a prediction-corrected visual predictive check and bootstrapping, both using 1000 simulated datasets and scripts available in Pirana.

Finally, we developed a rat PBPK model in PK-Sim v11.2 (Open Systems Pharmacology) to investigate the physiologic rationale behind the observed alterations in PK. A 17-compartment PBPK model representing virtual “organs” interconnected by blood flow was used for all simulations. The model was adapted from 2 human PBPK models: Kovar et al^19^ and an unpublished model developed to guide dosing in critically ill children. Rat-specific model parameters were obtained from 2 rat models published by Shao et al^35^ and Zhang et al.^36^ Tissue-specific organ partitioning coefficients were obtained from the rat models. Human models were used to determine the fraction of fentanyl metabolism for each specific P450 (CYP3A4/5 in humans). Including CYP3A4/5-specific metabolism allowed for the scaling of the model to rat and inclusion of the metabolite norfentanyl.

The rat fentanyl model was built using orthologs for human CYP3A4/5 (CYP3A18, CYP3A1/23, CYP3A62, and CYP3A9) and rat-specific physiology. Rat profiles within the modeling software did not include sex-specific physiology. Sex-specific physiological parameters were taken from the literature to create separate male and female rat individuals (Table 1).^19^^,^^35^, ^36^, ^37^, ^38^, ^39^, ^40^, ^41^, ^42^, ^43^ Size differences were accounted for within the software through calculated adjustments to organs based on inputted weight values. Additional changes were then made to reflect sex-specific differences that could not be accounted for based on size alone. Expression data for rat P450s were pulled from Bgee.org version 15.2.0 (Université de-Lausanne). Sprague-Dawley expression data were selected when possible. If not present, the mean value of post pubescent or adult values were used. P450 data were included for liver, kidney, and gastrointestinal tract. Sex-specific expression profiles were created to represent the difference in expression between male and female rats. Male and female individuals were created for model optimization using mean values for weights. Fentanyl metabolism was then divided among P450s based on rat microsomal in vitro studies.^15^^,^^44^^,^^45^ Sex-specific individual P450 V_max_ and K_m_ constants were estimated based on in vitro data and optimized using a Monte Carlo-based algorithm within PK-Sim. K_cat_ values were then calculated within the software based on individual P450 abundance and V_max_. CYP3A62 was included in initial models, but removed from subsequent models because of negligible contributions to fentanyl metabolism and model performance.^44^ Final parameter optimization was completed using a Monte Carlo-based algorithm within PK-Sim (Table 2).

**Table 1:** PBPK model parameters used for male and female individual rats Sex-specific physiologic parameters were adjusted based on published data^19^^,^^35^, ^36^, ^37^, ^38^, ^39^, ^40^

Parameter	Male	Female
Weight (g)	374	328
Venous blood (mL)	17.54	17.8
Arterial blood (mL)	7.63	7.8
Muscle (L)	0.02	0.01
Fat (L)	0.06	0.08
Hematocrit	0.44	0.4

**Table 2:** Fentanyl and norfentanyl PBPK model parameters

Parameter	Fentanyl	Norfentanyl
Physicochemical properties		
Lipophilicity (LogP)	3.95^a^	2.68^a^
Plasma binding protein	Albumin^19^	Albumin^19^
Fraction unbound (F_u_) (%)	10^a^	97^a^
Molecular weight (g/mol)	336.47^19^	232.32^19^
pKa	8.99^19^^,^^35^	10.00^19^^,^^35^
Distribution		
Partition coefficients	Rodgers and Rowland^19^	Schmitt^19^
Cellular permeabilities	Charge-dependent Schmitt^19^	PK-Sim Standard^19^
Metabolism and elimination		
GFR	1.0^19^	
Plasma clearance renal (mL/min per kg)		16.4^a^

CYP450 enzymes		

	Male	Female

*CYP3A1/23*		
K_m_ (*μ*M)	37^15^^,^^44^^,^^45^	37^15^^,^^44^^,^^45^
K_cat_ (L/min)	8.00^b^	1.00^b^
*CYP3A18*		
K_m_ (*μ*M)	37^15^^,^^44^^,^^45^	37^15^^,^^44^^,^^45^
K_cat_ (L/min)	1.86^b^	57.14^b^
*CYP3A9*		
K_m_ (*μ*M)	37^15^^,^^44^^,^^45^	37^15^^,^^44^^,^^45^
K_cat_ (L/min)	9.38^b^	0.16^b^

a Values were optimized within modeling software.

b K_cat_ was calculated in the modeling software using V_max_ and P450 abundance.

After optimization of fentanyl parameters, a norfentanyl compound was created, and integrated into the model as a metabolite. Parameters for norfentanyl were obtained from the Kovar et al and Zhang et al models.^19^^,^^36^ Male and female individuals were then created using mean values for weights across all injection days to capture changes in growth during the experimental timeframe. These individuals were used as the mean for virtual populations. Two populations were created: 1 female and 1 male. Each population contained 1000 virtual rats with variation in P450 enzyme expression of ± 62% was added to account for biological variation. Dosing time was included as the median time within the experimental window of approximately 4 hours. Model performance was then evaluated using a published rat fentanyl and norfentanyl PK study.^46^ A sensitivity analysis was then run in PK-Sim to determine the parameter importance of sex-specific differences to drug exposure (AUC). Variabilities were set to ±0.1, 0.5, 1, 2.5, and 10-fold variability.^35^ All sex-specific parameters in Table 1 were tested. Analyses and visualizations were completed with R Version 4.6.1 using tidyverse.^47^

## 3 Results

### 3.1 Study population

A total of 19 animals were included in the present study (9 females and 10 males), to ensure data for 7 male and 7 female rats were available for each time point. Animals were replaced in the study because of death following the first injection (*n* = 2), loss of arterial catheter patency during the study (*n* = 2), or other health issues during the study period (*n* = 1). Female rats were older (13.4 vs 8.9 weeks; *P* < .0001) and smaller (286 vs 343 g; *P* < .0001) than males at baseline.

### 3.2 Noncompartmental analysis of fentanyl and norfentanyl

Figure 1 depicts the concentration-time profiles of fentanyl and norfentanyl. Concentration-time profiles of fentanyl and norfentanyl were similar across injections, with no statistically significant changes across repeated doses. The t_1/2_ of fentanyl observed in this study (Supplemental Table 1) indicates that substantial drug accumulation is unlikely with the 72-hour dosing interval used in the multidose protocol. Additionally, no statistically significant differences in fentanyl area under the concentration-time curve to infinity (AUC_∞_) or maximum concentrations (C_max_) were observed between male and female animals (Fig. 2). In contrast to this finding, norfentanyl AUC_∞_ and C_max_ were lower (*P* < .0001) in females than in males (Fig. 2). Female sex was also associated with a longer norfentanyl t_1/2_ than males (Fig. 2).

**Fig. 1:**
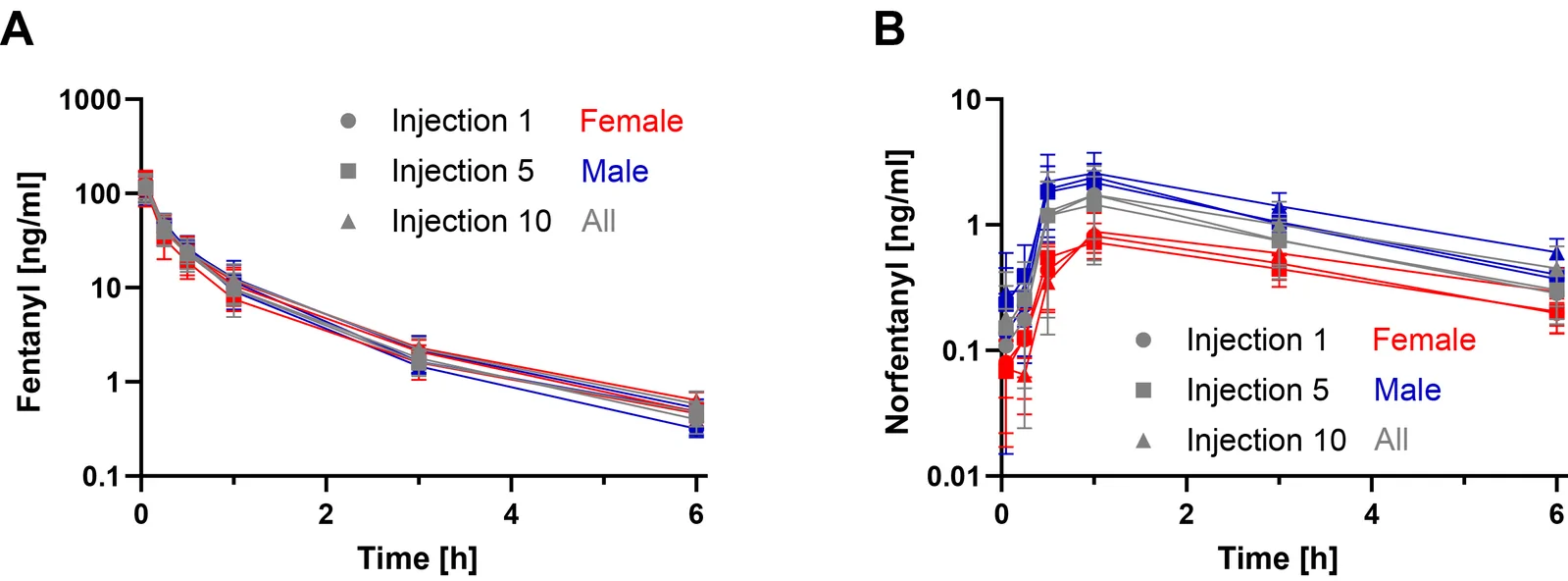
Plasma concentration-time profiles of both (A) fentanyl and (B) norfentanyl. Data points represent mean (SD) concentrations from 14 (all, in gray) or 7 (female, in red; males, in blue) rats. Data from the first injection are denoted with a filled circle, the fifth injection is marked with a filled square, and the tenth injection is depicted with a filled triangle.

**Fig. 2:**
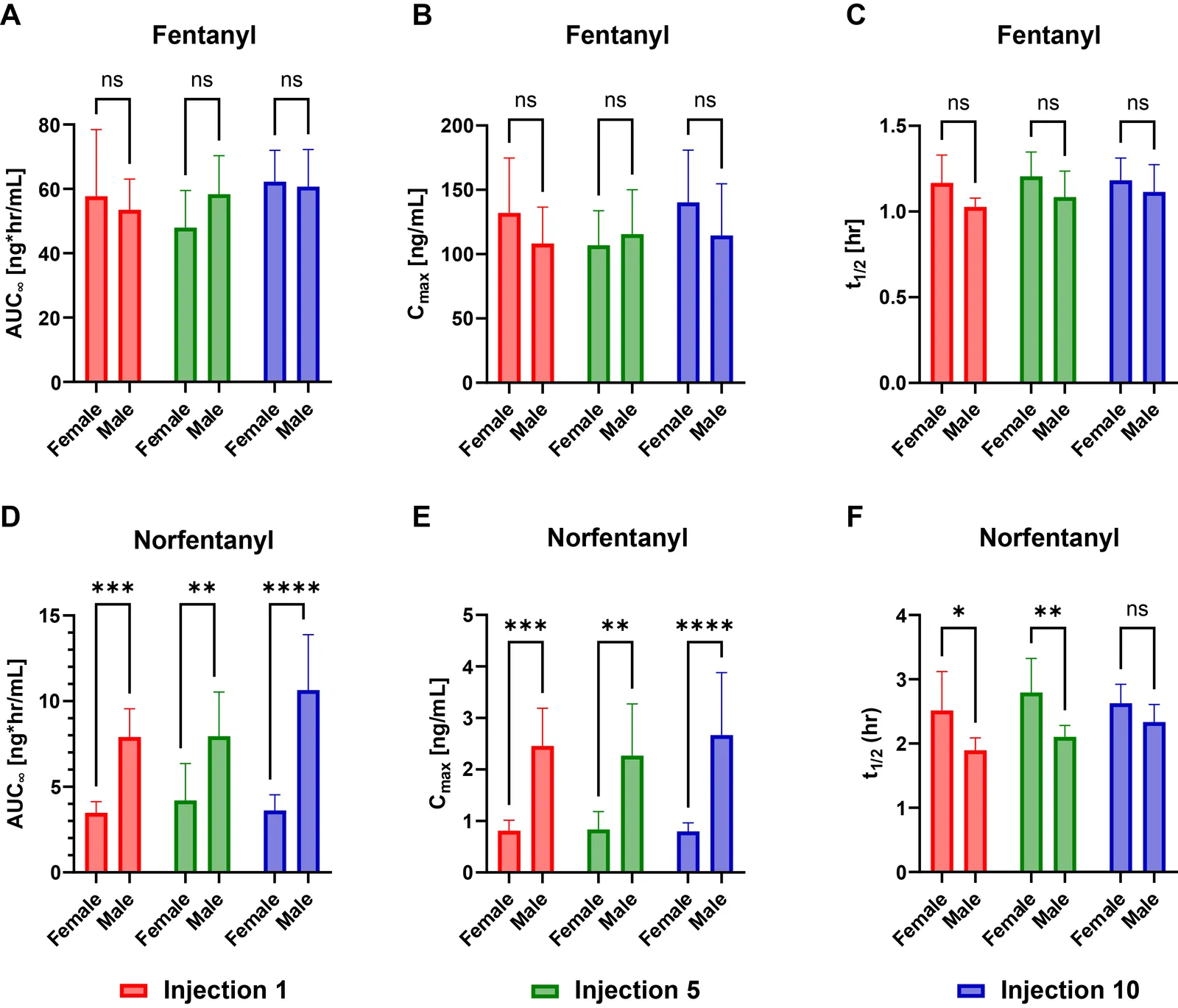
(A–C) Fentanyl and (D–F) norfentanyl AUC_∞_, C_max_, and t_1/2_, respectively, separated by sex and injection number. Data represent mean (SD) concentrations from 7 rats. Two-way ANOVA with Tukey multiple comparison tests were used for statistical analyses: ∗ .05 ≥ *P* > .01; ∗∗ .01 ≥ *P* > .001; ∗∗∗ .001 ≥ *P* > .0001; ∗∗∗∗ *P* ≤ .0001.

### 3.3 Population PK analysis of fentanyl and norfentanyl

We then constructed a population PK model to identify the PK process underlying the sex-associated differences in norfentanyl exposure, as well as to assess the sex-based weight and age differences in the study animals. The final population PK model schematic and code are included in the Supplemental Fig. 1 and Supplemental Material. Fentanyl PK was best described using a two-compartment model, whereas norfentanyl PK was best described with a one-compartment model, both parameterized using clearance and volume. Norfentanyl production was driven via Michaelis-Menten kinetics, as this approach yielded an extremely significant reduction in OFV relative to the use of a linear clearance model (ΔOFV = −161, *P* < .0001). Interindividual variability was modeled using η values on fentanyl clearance (CL_Fent_), the maximum velocity of norfentanyl production (V_max_), and norfentanyl clearance (CL_Nor_); attempts to estimate η values on other parameters (including volumes) resulted in poor performance and instability in the model. Both fentanyl and norfentanyl used proportional error models.

Covariate analysis identified sex (*P* = .001) and injection number (*P* = .004) on CL_Fent_, sex on V_max_ (*P* < .0001), and sex on CL_Nor_ (*P* = .004) as model significant. Of note, although injection number was categorical during covariate modeling, the parameter estimate describing the difference between injection 1 and 5 was minimal, so this covariate was dichotomized (injection 10 vs injection 1 and 5) prior to further analysis with the model, with minimal change in the OFV (ΔOFV = 0.774). These results indicated that CL_Fent_ is 16% slower in females relative to males and 8% slower after the 10th injection as compared with injection 1 or 5. Production of norfentanyl (V_max_) is reduced by 76% and CL_Nor_ is reduced by 45% in females relative to males. Final parameter-covariate equations and parameter estimates are included in Table 3, in addition to results of the bootstrap analysis. The goodness-of-fit plots and visual predictive check (Supplemental Fig. 2) support model acceptance, as do the bootstrap results (Table 3), although there is some indication in these results of model instability.

**Table 3:** Final population PK model parameter estimates and bootstrap analysis results

Parameter	Estimate (%RSE)[Eta Shrinkage]	Bootstrap (95% CI)
CL_Fent_ (mL/h)	774 (5%)	771 (689, 858)
CL_Fent_:Sex	−0.158 (26%)	−0.157 (−0.238, −0.0787)
CL_Fent_:Injection	−0.0796 (54%)	−0.077 (−0.165, 0.00594)
V_Fent,Central_ (mL)	275 (9%)	274 (219, 332)
Q_Fent_ (mL/h)	232 (18%)	254 (102, 363)
V_Fent,Peripheral_ (mL)	326 (9%)	335 (251, 401)
K_m_ (ng/mL)	2.89 (17%)	3.19 (0.557, 5.22)
V_max_ (ng/mL per h)	78.8 (12%)	71.5 (10.0, 148)
V_max_:Sex	−0.764 (7%)	−0.761 (−0.881, −0.647)
CL_Nor_ (mL/h)	23.6 (11%)	21.0 (4.25, 42.9)
CL_Nor_:Sex	−0.445 (27%)	−0.434 (−0.728, −0.162)
V_Nor_ (mL)	10.4 (18%)	9.23 (2.84, 18.0)
ω_CL,Fent_^2^	0.00843 (20%) [9%]	0.00648 (0.00300, 0.0138)
ω_Vmax_^2^	0.0716 (37%) [11%]	0.0536 (0.00794, 0.135)
ω_CL,Nor_^2^	0.0602 (65%) [24%]	0.0387 (−0.00988, 0.130)
Prop. error, fentanyl	0.112 (17%) [2%]	0.107 (0.0792, 0.145)
Prop. error, norfentanyl	0.261 (11%) [3%]	0.261 (0.206, 0.316)

CL_Fent_, fentanyl clearance; CL_Fent_, Sex, sex covariate effect on fentanyl clearance; CL_Fent_, injection, injection covariate effect on fentanyl clearance; V_Fent,Central_, fentanyl central compartment volume; Q_Fent_, fentanyl intercompartmental clearance; V_Fent,Peripheral_, fentanyl peripheral compartment volume; K_m_, Michaelis constant; V_max_, maximum enzyme reaction rate; V_max_, Sex, sex covariate effect on maximum enzyme reaction rate; CL_Nor_, norfentanyl clearance; CL_Nor_, sex, sex covariate effect on norentanyl clearance; V_Nor_, norfentanyl volume; ω_CL,Fent_^2^. interindividual variability on fentanyl clearance; ω_Vmax_^2^, interindividual variability on maximum enzyme reaction rate; ω_CL,Nor_^2^, interindividual variability on norfentanyl clearance.

### 3.4 PBPK analysis of fentanyl and norfentanyl

A rat fentanyl and norfentanyl PBPK model was developed to identify a physiological rationale for the observed sex-specific differences in fentanyl/norfentanyl. Sex-specific differences were incorporated into the virtual populations and model parameters (Tables 1 and 2). Male and female model simulations were run separately for fentanyl and norfentanyl by dosing regimen (Fig. 3, Supplemental Fig. 3). Additional model evaluation was performed by comparing model estimates with a published PK study in male rats (Supplemental Fig. 4).^46^ Model performance varied by sex and study day. Fentanyl model performance was best on day 1 with 93% of male and 97% of female observed data within the prediction interval. By day 31‒36, about 80% of male observed data fell within the prediction interval; however, model performance remained high in females, with 93% of observed data within the prediction interval. Female norfentanyl model predictions performed best and remained above 90% for the entirety of the experimental timeframe. Male norfentanyl model performance ranged from 82% of observed data within the prediction interval on day 1 to 60% of observed data falling within the prediction interval by days 31‒36. External model evaluation using published PK data from male Wistar rats demonstrated good agreement for norfentanyl (Supplemental Fig. 4).

**Fig. 3:**
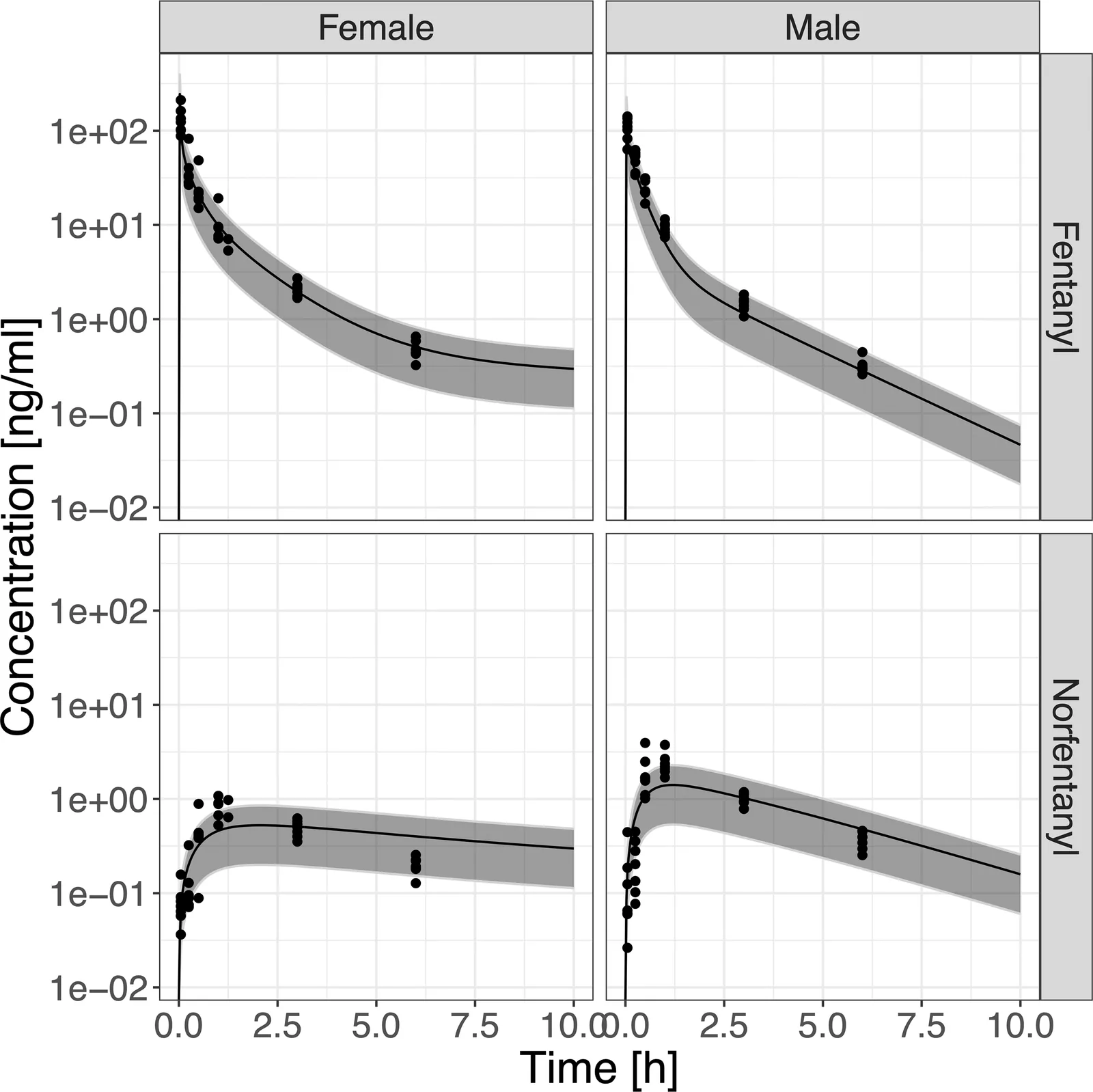
Fentanyl and norfentanyl PBPK model evaluations for male and female rats on day 1. Black circles represent individual plasma concentrations, the black line is the median model concentration prediction, and the gray shaded area represents the 90% confidence interval.

A sensitivity analysis was performed for male and female PBPK model simulations to identify key sex-specific physiologic parameters important in fentanyl PK (Supplemental Fig. 5). A 10-fold increase in sex-specific parameter values was used to assess the relative impact on the PK of fentanyl and norfentanyl. Muscle volume, fat volume, hematocrit, and venous blood volume were all identified as important sex-specific parameters for total clearance, C_max_, and AUC_inf_ (Supplemental Fig. 5). In both sexes, fentanyl PK was sensitive to alterations in muscle volume, venous blood volume, hematocrit, and fat volume. However, muscle volume and hematocrit had a greater effect in males, with fat volume more pronounced in females. For norfentanyl, hematocrit had the largest effect on PK in both males and females. Overall, more sex-specific physiological parameters were associated with the PK of fentanyl than that of norfentanyl.

## 4 Discussion

In this study, we evaluated the effect of repeated, high-dose fentanyl exposures on its metabolism using a rodent model. The noncompartmental analysis revealed no significant differences in fentanyl metabolism over 10 injections. However, there were sex-specific differences in its metabolism as norfentanyl AUC_∞_ and C_max_ were lower, whereas norfentanyl t_1/2_ was longer in the females compared with males. Constructing a population PK model, we found that fentanyl followed best a two-compartment model whereas norfentanyl was best described by a one-compartment model. The covariate analysis confirmed the significant influence of sex on fentanyl metabolism, revealing that fentanyl clearance, norfentanyl clearance, and norfentanyl production were significantly slower in females. Additionally, overall fentanyl clearance was significantly slower after the 10th exposure. The PBPK model performed well for fentanyl but less accurately for norfentanyl. The sensitivity analysis identified hematocrit and plasma protein concentrations as key physiological factors affecting fentanyl and norfentanyl kinetics in both sexes. For fentanyl, fat volume was positively correlated with drug exposure in females, whereas in males, greater muscle volume increased fentanyl clearance.

We found sex-specific differences in fentanyl metabolism, which might be explained by species-specific metabolism and sex-specific expression of P450 enzymes. In humans, fentanyl is primarily metabolized to norfentanyl by CYP3A4.^15^ However, in rats the major P450 isoform catalyzing fentanyl oxidation is not its human ortholog CYP3A9, but rather CYP3A1/2.^44^^,^^48^ Whereas CYP3A9 is induced by estrogen and has significantly higher gene expression in female hepatic samples than in males, CYP3A2 is a male-specific isoform of P450.^23^^,^^49^ For these reasons, we observed an increased fentanyl clearance and faster norfentanyl production in males compared with females. In contrast to our findings, Ohtsuka et al^31^ did not identify statistically significant sex differences in fentanyl PK following intravenous administration in rats but reported a nonsignificant trend toward higher fentanyl clearance in females. The reasons for this discrepancy may relate to differences in study design, including the lower fentanyl dose, the small sample size, and the use of a one-compartment PK model. Furthermore, the present study included norfentanyl measurements and used population and PBPK modeling approaches, which may have increased sensitivity to detect sex-dependent differences in fentanyl metabolism and clearance. Interestingly, overall fentanyl exposure did not significantly differ between sexes, suggesting that females may eliminate a greater proportion of fentanyl through nonhepatic pathways. Previous studies have focused on sex-specific differences in fentanyl PD rather than PK.^24^^,^^25^ Although findings vary depending on the endpoint assessed, most studies reported no significant sex differences in fentanyl-induced analgesia, respiratory depression, or sedation.^28^^,^^34^^,^^50^^,^^51^ However, most studies primarily evaluated peak PD effects, whereas the principal sex-dependent PK difference observed in the present study was fentanyl clearance, which may have a greater influence on the duration rather than the magnitude of fentanyl effects. Nevertheless, Bartok et al^50^ did not identify significant sex differences in either the magnitude or time course of fentanyl-induced antinociception in rats. The observed sex differences in norfentanyl formation are also unlikely to translate into PD effects, as norfentanyl is considered pharmacologically inactive. Taken together, the available PD literature in rodents is heterogeneous and does not support a consistent pattern of sex-dependent differences in fentanyl effects. This is broadly consistent with our findings, which demonstrate sex differences in fentanyl metabolism but not in overall fentanyl exposure.

The changes in fentanyl metabolism with repeated exposures were limited, but we observed a decreased fentanyl clearance after the 10th exposure. This is in line with a previous study that demonstrated a protracted renal clearance of fentanyl in persons with opioid use disorder.^21^ A potential mechanism might be the downregulation of P450 enzyme expression. Opioids themselves can influence P450 expression, as it was shown that morphine downregulates CYP3A1, which is known to be involved in fentanyl metabolism in rats.^52^ Additionally, hypoxia-inducible factor (HIF) activity has been linked to the suppression of CYP3 family expression.^53^ In our model, the rats were repeatedly hypoxic, which causes HIF expression, and fentanyl itself activates HIF in a *μ*-opioid receptor (MOR)–dependent manner. Therefore, HIF-associated downregulation might explain the reduced fentanyl clearance with repeated exposures.^54^ However, the fentanyl tolerance observed clinically in individuals with opioid use disorder indicates that these effects may be less relevant in humans, or are outweighed by adaptations in MOR signaling.^55^ In opioid-naïve individuals, Egan et al^56^ reported no meaningful changes in fentanyl PK following repeated, short-interval transmucosal fentanyl administration. Despite important differences in study design, including species, route of administration, dosing regimen, and the absence of complete washout between doses, these findings are consistent with our results. Although both male and female participants were included in that study, sex-specific PK analyses were not reported, limiting direct comparison with the sex-dependent findings observed in the present study.

Consistent with previous reports, fentanyl PK was best captured by a two-compartment model, reflecting its high lipophilicity, rapid distribution into well perfused tissues, and prolonged redistribution from peripheral compartments.^57^ As a consequence, the t_1/2_ observed in our study aligns with values reported in two-compartment analyses but exceeds those derived from one-compartment models, which underestimate the slower terminal elimination phase.^12^^,^^31^^,^^58^ Our PBPK model underlines the role of redistribution from peripheral compartments such as muscle and fat as key determinants of fentanyl clearance. We found that muscle volume determined total body clearance in males, whereas fat volume increased overall drug exposure in females, which is presumably attributable to sex differences in body composition.^59^ Since redistribution from muscle occurs more rapidly than from fat, not only the previously described sex differences in P450 enzyme expression but also the higher percentage of body fat in females may contribute to the longer fentanyl clearance observed in this group.^60^

### 4.1 Limitations

In the present study, we only evaluated PK following intravenous fentanyl administration. We focused on this route of administration because it is one of the predominant routes in illicit fentanyl use and is most commonly associated with overdose deaths.^33^^,^^61^ As we were interested in the effects of repeated fentanyl overdose, we chose a bolus dose of 100 *μ*g/kg, which was associated with a lethality of approximately 10% in opioid-naïve animals.^28^ Consequently, 2 animals died after the first fentanyl injection, and a survivorship bias cannot be excluded, as their PK profiles may have differed from those of the surviving animals included in the analysis. To ensure a full washout of fentanyl between exposures and give the animals enough time to recover between doses, we administered fentanyl twice per week with at least 72 hours between exposures.

Additionally, our findings are derived from a rat model designed to mimic repeated fentanyl overdoses. Given the limitations of animal models in predicting human toxicity and known species-specific differences in fentanyl metabolism, the direct translatability of these results to humans may be limited.^62^ Furthermore, only norfentanyl was quantified in plasma in the present study. Although norfentanyl represents the major metabolic pathway of fentanyl, neither other metabolites nor urinary excretion was assessed.^14^^,^^15^ Therefore, the contribution of alternative metabolic pathways or renal clearance to the observed sex differences could not be determined.

### 4.2 Conclusion

In this study, we evaluated the impact of repeated high-dose fentanyl administration on its PK. In addition to a modest reduction in fentanyl clearance after the final exposure, we identified pronounced sex differences in fentanyl metabolism. Female animals exhibited slower clearance of both fentanyl and norfentanyl, and they produced overall less norfentanyl than males. These findings highlight the importance of sex-specific analysis and warrant future studies to evaluate potential clinical implications.

## Conflict of interest

The authors declare no conflicts of interest.
