# Supplementary material for: Pharmacokinetics of repeated, high-dose fentanyl administration in rats
Source: Drug Metab Dispos. 2026 Aug 17;54(9):100390. doi: 10.1016/j.dmd.2026.100390 (PMC13670244; doi:10.1016/j.dmd.2026.100390)
Supplement: Supplementary file 1 [file mmc1.docx]

**Supplemental Material**

**Table of content:**

Supplemental Table 1:	Fentanyl and norfentanyl PK parameters determined via non-compartmental analysis

Supplemental Material 1:	Population PK model code

Supplemental Figure 1:	Population model schematic

Supplemental Figure 2:	Population PK model diagnostics

Supplemental Figure 3:	Fentanyl and norfentanyl PBPK model evaluations for male and female rats 0 – 1 hours after dosing on Day 1

Supplemental Figure 4:	Fentanyl and norfentanyl PBPK model performance with published rat PK data

Supplemental Figure 5:	Model sensitivity analyses to identify sex-specific parameters

***Supplemental Table 1:*** *Mean (%coefficient of variation, %CV) fentanyl and norfentanyl PK parameters (t_1/2_, C_max_, and AUC_∞_) determined via non-compartmental analysis.*

|  | ***Injection 1*** | | | ***Injection 5*** | | | ***Injection 10*** | | |
| --- | --- | --- | --- | --- | --- | --- | --- | --- | --- |
| **Fentanyl** | **Total** | **Female** | **Male** | **Total** | **Female** | **Male** | **Total** | **Female** | **Male** |
| *C_max_*  *(ng/mL)* | 120 (30.7%) | 132 (32.4%) | 108 (26.1%) | 111 (27.0%) | 107 (25.1%) | 116 (29.8%) | 127 (32.3%) | 140 (29.0) | 115 (35.1%) |
| *AUC_∞_*  *(ng*hr/mL)* | 55.6 (28.2%) | 57.7 (35.9%) | 53.5 (17.8%) | 53.2 (23.5%) | 48.0 (24.0%) | 58.4 (20.4%) | 61.5 (16.7%) | 62.3 (15.6%) | 60.7 (19.0%) |
| *t_1/2_*  *(hr)* | 1.10 (12.4%) | 1.17 (13.9%) | 1.03 (4.9%) | 1.14 (13.4%) | 1.21 (11.7%) | 1.08 (13.9%) | 1.15 (12.5%) | 1.18 (11.0%) | 1.11 (14.2%) |
| **Norfentanyl** | **Total** | **Female** | **Male** | **Total** | **Female** | **Male** | **Total** | **Female** | **Male** |
| *C_max_*  *(ng/mL)* | 1.63 (61.1%) | 0.812 (24.8%) | 2.46 (29.9%) | 1.55 (66.8%) | 0.833 (41.6%) | 2.27 (44.2%) | 1.73 (73.7%) | 0.800 (20.6%) | 2.67 (45.5%) |
| *AUC_∞_*  *(ng*hr/mL)* | 5.69 (45.6%) | 3.48 (18.9%) | 7.90 (21.0%) | 6.08 (49.2%) | 4.21 (51.0%) | 7.95 (32.4%) | 7.13 (60.3%) | 3.62 (25.1%) | 10.6 (30.4%) |
| *t_1/2_*  *(hr)* | 2.21 (24.4%) | 2.52 (24.1%) | 1.90 (10.2%) | 2.45 (21.2%) | 2.79 (18.9%) | 2.11 (8.3%) | 2.48 (12.6%) | 2.63 (11.2%) | 2.33 (11.7%) |

***Supplemental Material 1:*** *Population PK model code*

;; 2. Description: FENT 2compCL to NFent 1compCL MM

$PROBLEM	RUN# FentMet

$INPUT	C ID TIME AMT DV MDV EVID CMT ADDL II SEX WTG WTKG AGE INJ STRAT

$DATA	Nor_Fent_NM.csv IGNORE=C

$SUBROUTINE ADVAN13 TOL=4

$MODEL	NPAR=9 NCOMP=3

COMP=(CENTRAL,DEFDOSE,DEFOBS) COMP=(PERIPHERAL)

COMP=(METABOLITE)

$PK

;;; CLINJ-DEFINITION START

IF(INJ.EQ.1) CLINJ = 1

IF(INJ.EQ.5) CLINJ = 1

IF(INJ.EQ.10) CLINJ = ( 1 + THETA(12))

;;; CLINJ-DEFINITION END

;;; CLSEX-DEFINITION START

IF(SEX.EQ.1) CLSEX = 1 ; Most common

IF(SEX.EQ.0) CLSEX = ( 1 + THETA(9))

;;; CLSEX-DEFINITION END

;;; CL-RELATION START

CLCOV=CLSEX*CLINJ

;;; CL-RELATION END

TVCL=THETA(1)

TVCL = CLCOV*TVCL

CL=TVCL*EXP(ETA(1))

TVV1=THETA(2)

V1=TVV1;*EXP(ETA(2))

TVQ=THETA(3)

Q=TVQ

TVV2=THETA(4)

V2=TVV2

TVKM=THETA(5)

KM=TVKM

;;; VMAXSEX-DEFINITION START

IF(SEX.EQ.1) VMAXSEX = 1 ; Most common

IF(SEX.EQ.0) VMAXSEX = ( 1 + THETA(11))

;;; VMAXSEX-DEFINITION END

;;; VMAX-RELATION START

VMAXCOV=VMAXSEX

;;; VMAX-RELATION END

TVVMAX=THETA(6)

TVVMAX = VMAXCOV*TVVMAX

VMAX=TVVMAX*EXP(ETA(2))

;;; NCLSEX-DEFINITION START

IF(SEX.EQ.1) NCLSEX = 1 ; Most common

IF(SEX.EQ.0) NCLSEX = ( 1 + THETA(10))

;;; NCLSEX-DEFINITION END

;;; NCL-RELATION START

NCLCOV=NCLSEX

;;; NCL-RELATION END

TVNCL=THETA(7)

TVNCL = NCLCOV*TVNCL

NCL=TVNCL*EXP(ETA(3))

TVV3=THETA(8)

V3=TVV3

S1=V1

S3=V3*(336.5/232.3) ; Ratio of fentanyl:norfentanyl

;Calculate time after dose (TAD)

IF (AMT.GT.0) THEN

TDOS=TIME

TAD=0.0

ENDIF

IF (AMT.EQ.0) TAD=TIME-TDOS

$DES

C1=A(1)/V1 ; Fentanyl central compartment concentration

C3=A(3)/V3 ; Norfentanyl concentration

DADT(1) = -(CL/V1)*A(1) + (Q/V2)*A(2) - (Q/V1)*A(1)

DADT(2) = (Q/V1)*A(1) - (Q/V2)*A(2)

DADT(3) = (VMAX*C1)/(KM+C1) - (NCL/V3)*A(3)

$ERROR

CC1=A(1)/V1 ; Fentanyl central compartment concentration

CC3=A(3)/V3 ; Norfentanyl concentration

DEL=0

IF (F.LE.0.0001) DEL=1

IF(CMT.EQ.1) THEN ; error model for fentanyl concentrations

IPRED=CC1

W=IPRED+DEL

IRES=DV-IPRED

Y=F*(1+ERR(1)) ; Proportional error, Fentanyl

ELSE ; error model for norfentanyl concentrations

IPRED=CC3

W=IPRED+DEL

IRES=DV-IPRED

Y=F*(1+ERR(2)) ; Proportional error, Norfentanyl

ENDIF

$ESTIMATION METHOD=1 INTERACTION PRINT=10 MAX=9999 SIG=1 MSFO=FentMet.msf NOABORT

$THETA

(0, 785) ; ([CL])

(0, 276) ; ([V1])

(0, 232) ; ([Q])

(0, 328) ; ([V2])

(0, 2.93) ; [KM]

(0, 79.1) ; [VMAX]

(0, 23.6) ; [NCL]

(0, 10.4) ; [V3]

(-1, -0.157,5) ; CLSEX1

(-1, -0.448,5) ; NCLSEX1

(-1, -0.763,5) ; VMAXSEX1

(-1, -0.0928,5) ; CLINJ10

$OMEGA

0.00873 ; [P] omega(1,1)

0.0725 ; [P] omega(2,2)

0.0615 ; [P] omega(3,3)

$SIGMA

0.112 ; [P] sigma(1,1)

0.261 ; [P] sigma(2,2)

$COV

***Supplemental Figure 1:*** Population model schematic

***Supplemental Figure 2:*** *Goodness of fit plots and visual predictive checks for the population PK model. Goodness of fit plots for fentanyl are shown in a, while the same plots for norfentanyl are shown in b. Visual predictive checks for fentanyl and norfentanyl are in panel c.*

*a*

*b*

*c*

***Supplemental Figure 3:*** *Fentanyl and norfentanyl PBPK model evaluations for male and female rats 0 – 1 hours after dosing on Day 1. Black circles represent individual plasma concentrations, the black line is the median model concentration prediction, and the grey shaded area represents the 90% confidence interval.*

***Supplemental Figure 4:*** *Fentanyl and norfentanyl PBPK model performance with published rat PK data. A 10ug/kg IV bolus dose of fentanyl was administered to 18 – 20 week old Wistar male rats.^1^ Black circles are published PK data, the solid black line is the median predicted concentration, and the grey shaded area is the 90% prediction interval.*

***Supplemental Figure 5:*** *Model sensitivity analyses to identify sex-specific parameters that may affect fentanyl and norfentanyl AUC, clearance, and maximum concentration (C_max_). Blue boxes represent male and pink female model parameters.*

**References**

1.	Stach P, Skowron K, Rojek S, Cios A, Wesołowska A, Huestis MA, et al. Pharmacokinetics of Fentanyl and Norfentanyl in a Rat Model of Fixed-Volume Hemorrhagic Shock. European Journal of Drug Metabolism and Pharmacokinetics. 2026.
